# Down-Regulation of *Serum/Glucocorticoid Regulated Kinase 1* in Colorectal Tumours Is Largely Independent of Promoter Hypermethylation

**DOI:** 10.1371/journal.pone.0013840

**Published:** 2010-11-05

**Authors:** Francesca Lessi, Andrew Beggs, Mariagrazia de Palo, Marcello Anti, Raffaele Macarone Palmieri, Simona Francesconi, Vito Gomes, Generoso Bevilacqua, Ian Tomlinson, Stefania Segditsas

**Affiliations:** 1 Molecular and Population Genetics Laboratory, Wellcome Trust Centre for Human Genetics, University of Oxford, Oxford, United Kingdom; 2 Presidio Ospedaliero Belcolle Viterbo, Viterbo, Italy; 3 Division of Surgical, Molecular and Ultrastructural Pathology, Department of Oncology, University of Pisa and Pisa University Hospital, Pisa, Italy; University of Insubria, Italy

## Abstract

**Background:**

We have previously shown that serum/glucocorticoid regulated kinase 1 (*SGK1*) is down-regulated in colorectal cancers (CRC) with respect to normal tissue. As hyper-methylation of promoter regions is a well-known mechanism of gene silencing in cancer, we tested whether the *SGK1* promoter region was methylated in colonic tumour samples.

**Methodology/Principal Findings:**

We investigated the methylation profile of the two CpG islands present in the promoter region of *SGK1* in a panel of 5 colorectal cancer cell lines by sequencing clones of bisulphite-treated DNA samples. We further confirmed our findings in a panel of 10 normal and 10 tumour colonic tissue samples of human origin. We observed CpG methylation only in the smaller and more distal CpG island in the promoter region of *SGK1* in both normal and tumour samples of colonic origin. We further identified a single nucleotide polymorphism (SNP, rs1743963) which affects methylation of the corresponding CpG.

**Conclusions/Significance:**

Our results show that even though partial methylation of the promoter region of *SGK1* is present, this does not account for the different expression levels seen between normal and tumour tissue.

## Introduction

The serum/glucocorticoid regulated kinase 1 (SGK1) is a recently identified member of the AGC family of serine/threonine kinases, which shares 50% similarity in its aminoacid sequence with other members of the family such as Akt/PKB, PKA and PKC-zeta [1]. *Sgk1* was originally identified as an immediate early gene induced in response to serum and glucocorticoid stimuli in rat mammary tumour cells [1]. A number of other stimuli such as growth pathway signalling factors [2], [3], cytokines [4], hormones [5], [6], [7], [8] and stress conditions [9], [10] have recently been found to induce activation of *Sgk1* transcription. Not surprisingly, over 40 potential transcription factor-binding sites have been predicted in the promoter region of *Sgk1* [11] and a number of functions have been attributed to this kinase in recent years.

Its best-studied roles are perhaps in the control of ion transport. In particular, SGK1 was shown to allow accumulation of the epithelial sodium transport channel ENaC by phosphorylating its ubiquitin ligase Nedd4-2 [12], thereby increasing Na^+^ re-absorption [13], [14]. SGK1 was also shown to regulate K^+^, Ca^2+^ and Cl^−^ channels and glucose transporters such as GLUT1 and SGLT1, probably resulting in regulation of cell volume and osmolarity, although these mechanisms are not well understood at present [14]. SGK1 was also found to affect the function of several kinases and transcription factors, including GSK3β [15], B-raf [16], components of the Erk signalling pathway [14], the cAMP responsive element (CREB) [17] and the forkhead transcription factor FKHRL1 (FOXO3a) [18]. Even though the outcome of these interactions is unclear in most cases, SGK1 has been implicated in the regulation of cell survival and apoptotic response [18], [19] and cell cycle progression [20]. Furthermore, its *C. elegans* homologue has been implicated in the control of development, stress response and longevity [21]. Not surprisingly then, *SGK1* expression was found to be deregulated in several tumour types (up-regulated in breast cancers [10], [19] and down-regulated in prostate cancers [22] and ovarian tumours [23]). However, no information is available at present on how modulation of *SGK1* expression in cancer is achieved.

We have previously shown that expression of *SGK1* is down-regulated in colorectal adenomas and carcinomas [24] in comparison to normal tissue. As hypermethylation of promoter regions has been shown to silence transcription [25] and to provide an alternative mechanism of inactivation of several genes [26], [27], [28], [29], we set out to investigate the methylation profile of the *SGK1* promoter region in colorectal cancer cell lines and in normal and tumour colonic tissue samples.

## Results

To investigate whether down-regulation of the *SGK1* transcript could be reversed in colorectal cancer cell lines, we treated the cells with serum and the corticosteroid dexamethasone, both of which have been previously reported to increase transcription of *Sgk1* [5], [15].

**Figure 1:**
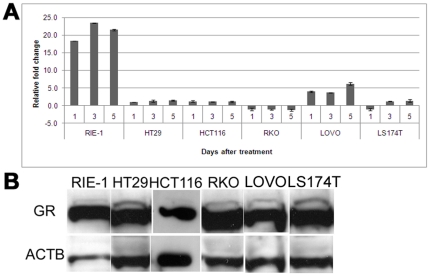
*SGK1* expression is not induced by glucocorticoids in colorectal cancer cells. *A*, fold change differences in *SGK1* expression levels upon Dexamethasone treatment, as measured by qRT-PCR relative to samples treated with vehicle only. The small intestinal rat cell line RIE-1 shows significantly increased expression levels (p<0.001), whereas no significant change is detected in the colorectal cancer cell lines HT29, HCT116, RKO and LS174T. Modestly increased levels (3.7 to 6.2-fold) of *SGK1* expression were seen in the colorectal cancer cell line LOVO. ***B***, representative western blot of the glucocorticoid receptor protein, showing strong expression in all lines tested. Actin beta (ACTB) was used as a loading control.

No significant increase in *SGK1* expression levels was found when the cells were treated with serum over a period of 5 days, after serum starvation (data not shown). Upon dexamethasone treatment, no significant increase in *SGK1* expression was seen in HT29, HCT116, RKO or LS174T cells after 1, 3 or 5 days, compared to untreated control (t-test p-values between 0.1 and 0.3). The CRC cell line LOVO showed modestly increased levels of *SGK1* expression (3.7 to 6.2-fold), while the RIE-1 cell line, which was used as a control as it is derived from normal small intestinal cells of rat origin, showed a prominent increase in *Sgk1* levels (average fold change = 21; t-test p-value = 1.2×10^−5^) as expected from previous reports (Fig. 1a).

To confirm that the difference seen was not due to a lack of glucocorticoid receptor (GR) in the CRC lines tested, we investigated GR protein levels by western blotting, and showed that the glucocorticoid receptor is abundantly expressed in all lines tested (Fig. 1b).

Given these results, we reasoned that one of the possible mechanisms responsible for the silencing of *SGK1* transcription could be hypermethylation of the promoter regions and we therefore set out to investigate the methylation profile of the *SGK1* promoter.

### CpG islands methylation profiles

Since the promoter region of human *SGK1* has not been functionally characterized, we based our investigation on the CpG islands identified by interrogation of the UCSC Human Genome Browser. Two CpG islands close to the transcription start site were identified and further investigated. Details are given in Materials and Methods and Figure S1.

DNAs extracted from HT29, HCT116, RKO, LOVO and LS174T colorectal cancer cell lines and the control proximal tubule kidney cell line HK2 were investigated by bisulphite conversion followed by cloning of the PCR products covering the CpG island regions and sequencing of the clones. Results were as follows.

#### CpG 1

Between 5 and 10 clones (median = 8) were sequenced for each cell line (average concordance between clones was 99%). No methylation was found in the CpGs in this region in any of the 5 CRC cell lines or in the control cell line HK2. HK2 cells treated with the CpG methyltransferase SssI were used as a positive control and consistently displayed methylation of all CpGs in the island.

#### CpG2

**Figure 2:**
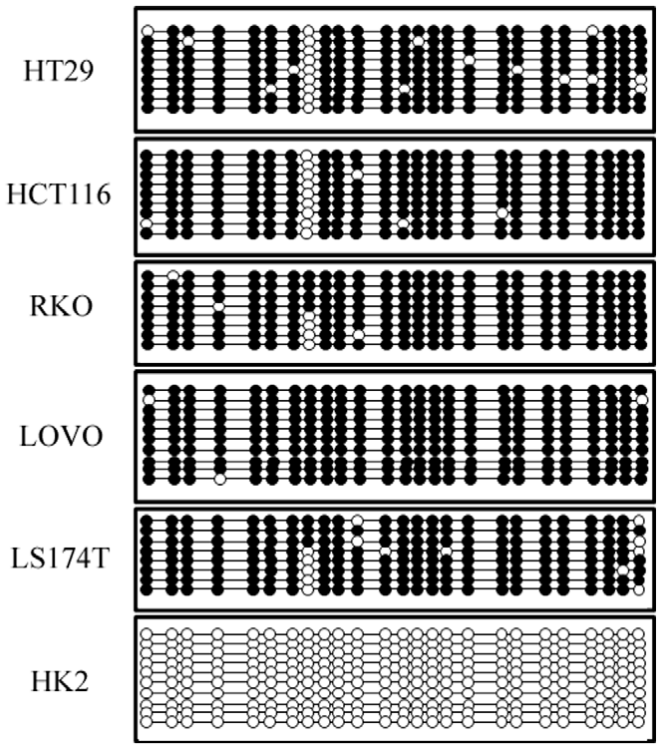
The CpG2 island is methylated in colorectal cancer cell lines. The dot diagrams report the methylation status at each of the 25 CpGs in the CpG2 island as assayed by sequencing multiple clones (between 8 and 10) from the colorectal cancer cell lines HT29, HCT116, RKO, LOVO and LS174T and the control renal cell line HK2 (filled circle = methylated; white circle = unmethylated).

All CRC cell lines tested, as well as the positive control, showed methylation of the CpGs in this region (between 8 and 10 clones were sequenced for each sample and average concordance between the clones was 97%), however the untreated control cell line HK2 did not (Figure 2).

To determine whether the methylation displayed by the CRC lines is due to their transformed nature, or whether the difference seen with respect to the control is attributable to tissue specificity, we investigated the methylation status of the CpGs in this region in a panel of DNAs extracted from 10 matched normal and tumour human colonic tissue samples. Down-regulation of the *SGK1* transcript was confirmed in all tumour samples by qRT-PCR (Figure S2).

**Figure 3:**
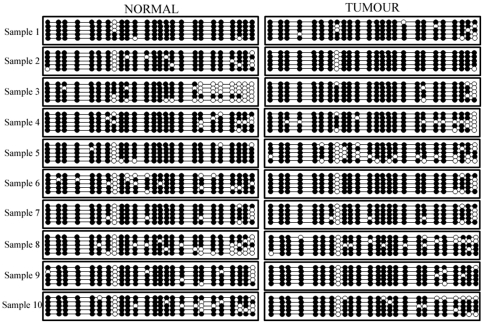
Both normal and tumour colonic tissue samples display methylation at sites in CpG2 island. The dot diagrams report the methylation status at each of the 25 CpGs in the CpG2 island as assayed by sequencing multiple clones (n = 5) from matched normal (left panels) and tumour (right panels) samples from 10 patients (filled circle = methylated; white circle = unmethylated).

**Figure 4:**
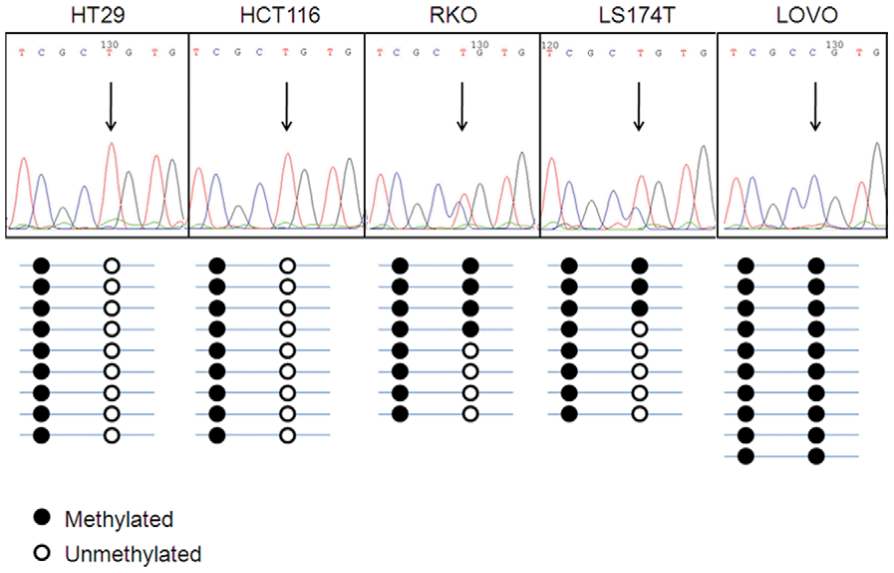
Correlation between genotype at SNP rs1743963 and methylation. Upper panels show representative images of the electropherograms obtained from sequencing the region surrounding the rs1743963 SNP in each of the CRC cell lines tested. Below each electropherogram is a summary of the methylation pattern found in the clones sequenced after bisulphite treatment of DNA (filled circle = methylated; white circle = unmethylated).

After bisulphite treatment and sequencing of a minimum of 5 clones for each sample, it was found that all tissue samples (normals and tumours) displayed almost complete methylation of the CpGs in this region (Figure 3). During our investigation, we found that the 8^th^ CpG in this smaller island (chr6:134497925) displayed a methylation pattern that varied among the cell lines tested and was found to be unmethylated in HT29 and HCT116 cells, hemimethylated in RKO and LS174T cells and methylated in LOVO cells. Upon interrogation of the UCSC Genome Browser, we found that the single nucleotide polymorphism (SNP) rs1743963 is present at this position and we therefore designed primers to the DNA sequence surrounding the SNP. Perfect correspondence between the genotype of the cell lines at this SNP and their methylation status for the 8^th^ CpG was found (Figure 4), consistent with the fact that this polymorphism disrupts the CpG by substituting the C allele with a T. We found HT29 and HCT116 cells to be homozygous for the T allele at this locus, RKO and LS174T cells to be heterozygous, while LOVO cells were found to be homozygous for the C allele, in accordance with the methylation patterns displayed.

### Demethylation treatment

**Figure 5:**
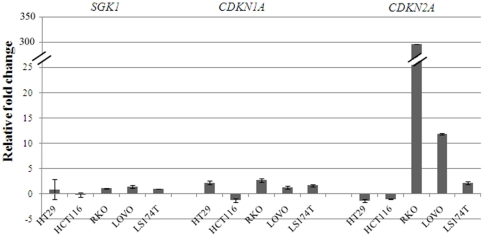
*SGK1* expression upon 5-AzaC treatment. The bar chart shows relative fold changes in *SGK1*, *CDKN1A* and *CDKN2A* expression as measured by qRT-PCR after treatment with the demethylating agent 5-AzaC. *SGK1* expression levels are not significantly affected by demethylating treatment (fold changes between −0.17 and 1.4), while expression of *CDKN1A* and *CDKN2A* is increased in most lines as expected (fold change increases 1.7 to 2.7 and 2.2 to 297 respectively).

To confirm the data observed, we treated the same CRC cell lines with 5-Aza-2′-deoxycytidine (5-AzaC), an inhibitor of DNA methylation, and assayed expression of *SGK1* in treated and untreated cells by qRT-PCR. As shown in Figure 5, treatment of the cells with 5-AzaC resulted in a very small and statistically non significant increase in *SGK1* transcription levels (fold change difference -1.5 to 1.4, t-test p-values≥0.06), in accordance with the finding that the main CpG island is unmethylated. Expression of *CDKN1A*, which was previously shown to increase with 5-AzaC treatment [30] and of *CDKN2A*, a gene known to be frequently methylated in colorectal cancers [31], were used as positive controls and showed increased transcription in most cell lines.

## Discussion

We have previously reported down-regulation of the *SGK1* transcript in colonic tumour tissue and colorectal cancer cell lines with respect to normal tissue [24]. In the present study we have shown that the down-modulation of *SGK1* in colorectal cancer cell lines cannot be relieved by stimulating the cells with serum or glucocorticoids, both of which are known inducers of *SGK1* transcription, suggesting that *SGK1* is actively repressed in colorectal cancer cells.

As hyper-methylation of promoter regions is a well known mechanism of gene inactivation and suppression of gene expression, we investigated whether it was responsible for the lowered expression of *SGK1* in colorectal tumours. The human *SGK1* promoter has not been functionally characterized, but two CpG islands are found close to the transcription start site (TSS). We initially investigated the methylation profile of all 151 CpGs contained within these two regions by sequencing several clones of the PCR products from bisulphite-treated DNA from 5 colorectal cancer cell lines (HT29, HCT116, RKO, LOVO and LS174T). No methylation was found at any of the 126 CpGs present in the island most proximal to the TSS, which we named CpG1, in any of the CRC cell lines tested. On the contrary, all CpG sites in the island found further upstream of the TSS (CpG2), were found to be methylated in all CRC cell lines, but not in the kidney-derived control cell line HK2. DNAs extracted from colonic tissues of both normal and tumour origin also displayed methylation of the CpG sites in this region, suggesting that the difference seen between the CRC cell lines and the control cell line HK2 is probably attributable to the different requirements for *SGK1* expression in different tissues. *SGK1* is known to play an important role in renal electrolyte excretion [32] and it is plausible that higher expression levels, or possibly the expression of different isoforms, are required in the kidneys than in the intestinal tract, which would explain the differences seen in the methylation patterns of the promoter region.

Further confirmation that down-regulation of *SGK1* in tumour samples is not highly dependent on promoter hypermethylation, was obtained by treating the CRC cell lines with 5-AzaC, an inhibitor of methylation. qRT-PCR results show that the increase in *SGK1* expression levels following demethylating treatment is small and non statistically significant, in accordance with the finding that the promoter region of the gene is unmethylated for the most part. *SGK1* transcript levels were not found to be highly increased even when 5-AzaC treatment was followed by dexamethasone treatment (data not shown), suggesting that stimulus-dependent induction of *SGK1* expression is also not greatly affected by methylation of the CpG2 island.

In addition we have found that the rs1743963 SNP affects the methylation status of the corresponding CpG. The significance of this data point is currently unknown. We could not find a direct correlation between the genotype of this SNP and levels of *SGK1* expression in the cell lines. However, it cannot be excluded that the presence of this SNP plays a role in the regulation of *SGK1* expression, for example through modulation of the binding of specific transcription factors in the region. As expression of *SGK1* is highly stimulus-dependent, such effects may only become apparent when the appropriate stimulus is applied to the cells.

In conclusion, our study shows that only the smallest of the two CpG islands present in the promoter region of *SGK1* is methylated in colonic tumour tissues and cell lines. However, this region was also found to be methylated in normal colonic tissue and therefore is unlikely to account for the differences in *SGK1* expression seen between normal and tumour tissue samples, which are instead most likely due to transcriptional repressors acting on the *SGK1* promoter. What these repressors are and how they are controlled remains to be defined.

## Materials and Methods

We have worked solely on anonymised samples. Study of these has been approved by Oxfordshire REC B 05/Q1605/66 and covers this study. Patient consent is not required since we cannot link molecular data back to the patient, however verbal informed consent was obtained from all patients anyway.

### Cell culture and dexamethasone treatment

The colorectal cancer (CRC) cell lines HT29, HCT116, RKO, LOVO and LS174T were sourced as previously published [33] and were grown in DMEM containing 10% foetal calf serum. The rat RIE-1 small intestinal cell line [34] was grown in RPMI medium with 10% foetal calf serum. The human proximal tubule kidney cell line HK2 was purchased from the American Type Culture Collection (Manassas, VA) and grown in keratinocyte medium supplemented with EGF and Bovine Pituitary Extract and was chosen as a control given the unavailability of human normal-like intestinal cell lines and the abundance of *SGK1* transcript in HK2 cells. All lines were maintained at 37°C and 5% CO_2_.

For dexamethasone treatment, cells were plated out and left to grow to about 80% confluence. Following serum starvation for 24 hours, fresh serum-free medium supplemented with 1 µM dexamethasone (Sigma) was added to the cells. Cells were assayed 1, 3 and 5 days after treatment.

### qRT-PCR

RNAs were extracted from cell pellets with the GenElute Mammalian Total RNA Miniprep Kit (Sigma-Aldrich), according to manufacturer's protocol. RNAs were converted to cDNA using the High Capacity cDNA Reverse Transcription Kit (Applied Biosystems), according to manufacturer's instructions. TaqMan Gene Expression Assays (Applied Biosystems) were used for *SGK1* (Hs00178612_m1; Rn00570285_m1), *CDKN1A* (Hs00355782_m1) and *GAPDH* (Hs99999905_m1; Rn99999916_s1), which was used as an endogenous control for normalization. qRT-PCR was performed on the ABI 7900HT (Applied Biosystems) according to manufacturer's instructions and data were analysed with the comparative Ct method, as described in Applied Biosystems's User Bulletin No. 2.

### GR western blot

Protein lysates were extracted from cultured cells, run on SDS-page and blotted using standard methods. Blotted membranes were blocked in 5% milk and incubated overnight with rabbit polyclonal anti-glucocorticoid receptor antibody (Abcam, ab3579-50, 1∶400). Then further washed and incubated with HRP-conjugated polyclonal goat anti-rabbit antibody (Dako, P0448) and detected by chemiluminscence (ECL kit, GE Healthcare). HRP-conjugated anti actin-beta antibody (Abcam, ab8226, 1∶10000) was used as loading control.

### DNA extraction (tissues and cell lines)

DNAs were extracted from fresh cell pellets using the DNeasy Blood and Tissue Kit (QIAGEN), according to manufacturer's protocol.

Normal and tumour tissue samples were obtained from 10 patients undergoing colic resection, after verbal informed consent had been taken. The samples were snap-frozen after resection. Tissue sections were cut from the frozen specimens and stained with haematoxylin and eosin. DNA was extracted from macrodissected areas of normal and tumour epithelium with the DNeasy Blood and Tissue Kit (QIAGEN), according to manufacturer's protocol.

### CpG methyltransferase treatment

DNA extracted from the HK2 cell line was treated with CpG methyltransferase SssI (NEB), according to manufacturer's protocol, purified by phenol/chloroform precipitation and used as a positive control. Untreated DNA from the same cell line was used as negative control.

### Bisulphite treatment

The DNA Methylation Kit (ZYMO RESEARCH) was used for bisulphite treatment of DNA samples, according to manufacturer's protocol. All reactions were performed in triplicates and pooled for increased yield.

### Choice of CpG regions, PCRs and primers

Two CpG islands were identified close to the transcription start site (TSS) of *SGK1* by interrogation of the UCSC Human Genome Browser. The CpG island most proximal to the TSS, which we named CpG1 (chr6:134495940-134496958), extends over 1019 bp and encompasses 126 CpGs. The second island (CpG2, chr6:134497537-134497756) is 220 bp long and contains 25 CpGs. Figure S1 represents the position of the CpG islands with respect to the TSS. Primers were designed to amplify the CpG island regions from bisulphite-treated DNA, taking care not to include any CpG in the primer sequence. The CpG1 region was split into three overlapping PCRs, due to its size and the difficulties in amplifying DNA after bisulphite treatment. Primer pairs were as follows:

CpG1.1 forward 5′-ttggttttggttaaaagtataaaaaa-3′

CpG1.1 reverse 5′-aataaaccaaacccccaac-3′

CpG1.2 forward 5′-ttgtttatgggggagatgt-3′

CpG1.2 reverse 5′-atccctacaaataccctctc-3′

CpG1.3 forward 5′-gggaggagggtgggagtt-3′

CpG1.3 reverse 5′-cccttaacaacctcaattttca-3′

CpG2 forward 5′-gggattgtgttagatttagtaggtaa-3′

CpG2 reverse 5′-tcaaaactaccccaaaacttcttaa-3′

Products were amplified using LA Taq (Takara Bio Inc). Conditions are available upon request.

### Cloning and sequencing

PCR products were cloned into the pGEMT vector, using the pGEMTeasy kit (Promega), according to manufacturer's protocol. DNA was isolated from between 5 and 10 clones in each occasion, using the QIAspin Miniprep Kit according to manufacturer's protocol, and used to PCR the inserted sequence with M13 primers (forward 5′-GTTTTCCCAGTCACGAC-3′, reverse 5′-CAGGAAACAGCTATGAC-3′). After clean up with the QIAquick PCR Purification Kit (Qiagen), the PCR products were used to perform a sequencing reaction, containing either the forward or the reverse M13 primer and Big Dye Terminator mix (Applied Biosystems). Sequencing reactions were run on an ABI3730 by the Zoology Department at Oxford University. Conversion of unmethylated cytosines was found to be 100% efficient. No new sequence data were generated, therefore no deposit was made into GenBank.

### 5-Aza-2′-deoxycytidine (5-AzaC) treatment

Cells were plated out into 6-well plates and left to grow to 70% confluency, at which point the medium was replaced with fresh medium containing 0.5 µM 5-AzaC (Sigma). After 24 hours the medium was replaced with fresh complete medium and the cells were grown for a further 48 hours before assaying. Cells treated with vehicle only served as controls.

## Supporting Information

Figure S1 Schematic representation of the CpG islands analyzed. The figure shows the position of the CpG islands analyzed with respect to the transcription start site (TSS). The filled (methylated) and white (unmethylated) circles represent each individual CpG within the island. The arrows indicate the direction of transcription (from right to left as the gene is on the negative strand).
(0.78 MB TIF)

Figure S2 SGK1 down-regulation in the primary tumour samples. The bar chart reports relative fold changes of SGK1 expression in the primary tumour samples analysed, compared to their matched normal tissue, as assayed by qRT-PCR. As expected, all samples showed down-regulation of SGK1 (between 3- and 951-fold).
(0.44 MB TIF)
