# Supplementary figures and images for: Down-Regulation of Serum/Glucocorticoid Regulated Kinase 1 in Colorectal Tumours Is Largely Independent of Promoter Hypermethylation
Source: PLoS One. 2010 Nov 5;5(11):e13840. doi: 10.1371/journal.pone.0013840 (PMC2974649; doi:10.1371/journal.pone.0013840)

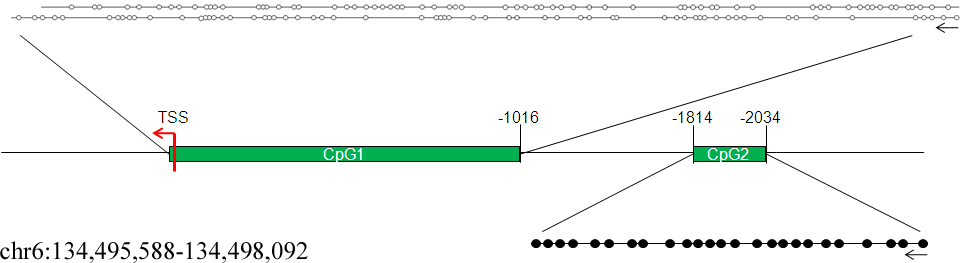

Supplement: Figure S1 — Schematic representation of the CpG islands analyzed. The figure shows the position of the CpG islands analyzed with respect to the transcription start site (TSS). The filled (methylated) and white (unmethylated) circles represent each individual CpG within the island. The arrows indicate the direction of transcription (from right to left as the gene is on the negative strand). (0.78 MB TIF) [file pone.0013840.s001.tif]

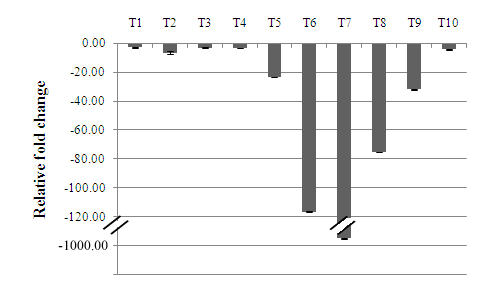

Supplement: Figure S2 — SGK1 down-regulation in the primary tumour samples. The bar chart reports relative fold changes of SGK1 expression in the primary tumour samples analysed, compared to their matched normal tissue, as assayed by qRT-PCR. As expected, all samples showed down-regulation of SGK1 (between 3- and 951-fold). (0.44 MB TIF) [file pone.0013840.s002.tif]
